# Posterior hemivertebra resection without internal fixation in the treatment of congenital scoliosis in very young children

**DOI:** 10.3389/fsurg.2022.1018061

**Published:** 2023-01-06

**Authors:** Bing Xia, Hongqian Wang, Yingmei Dong, Fuyun Liu, Wenjing Wang, Weiming Hu, Feipeng Wang, Fengqun Ma, Kai Wang

**Affiliations:** Department of Pediatric Orthopedics, The Third Affiliated Hospital of Zhengzhou University, Zhengzhou, China

**Keywords:** spinal surgery, hemivertebra resection, young children, x-ray, computer tomography

## Abstract

**Objective:**

To retrospectively analyze the feasibility and efficacy of posterior hemivertebra resection without internal fixation in the treatment of congenital scoliosis in very young children.

**Methods:**

Sixteen cases of very young children with congenital scoliosis treated at our hospital from April 2000 to July 2019 were collected, including 8 cases of each sex, all of whom had type I/III congenital scoliosis and were operated on at a median (interquartile range) of 9.00 (7.75) months (range, 0.5–48 months) of age. All cases underwent posterior hemivertebra resection without internal fixation and wore orthopedic braces or plaster undershirts for more than six months after surgery, with a mean follow-up of 94.31 ± 65.63 months (range, 36–222 months).

**Results:**

Coronal plane: the preoperative Cobb angle for the segmental curve was 39.50 ± 9.70° compared to postoperative (19.19 ± 8.56°) and last follow-up (14.94 ± 12.11°) (both *P *< 0.01); the preoperative Cobb angle for the main curve was 34.19 ± 14.34° compared to postoperative (17.00 ± 11.70°) and last follow-up (17.56 ± 16.31°) (both *P *< 0.01); the preoperative Cobb angle of the proximal compensated curve was 14.88 ± 9.62° compared to postoperative (7.88 ± 4.66°) and last follow-up (8.38 ± 8.36°) (both *P *< 0.05); and the preoperative Cobb angle of the distal compensated curve was 13.50° (10.50°) (range, 4°–30°) compared with postoperative 4.50° (9.25°) (range, −3° to 25°) and final follow-up 5.50° (9.50°) (range, −3° to 33°) (both *P* < 0.01). Sagittal plane: the difference in the preoperative Cobb angle was 10.00° (14.00°) (range, −31° to 41°) for segmental kyphosis compared to postoperative 14.00° (24.50°) (range, −6° to 46°) and last follow-up 17.00° (22.55°) (range, −40° to 56°), and these were not statistically significant (both *P *> 0.05). There was a tendency for the thoracolumbar kyphosis to worsen and the lumbosacral kyphosis to improve during the follow-up period.

**Conclusion:**

Posterior hemivertebra resection without internal fixation is a feasible treatment for type I/III congenital scoliosis in very young children, but the correction of the sagittal deformity of the thoracolumbar spine is not satisfactory, and postoperative external fixation may require further improvement.

## Introduction

Congenital scoliosis is a congenital abnormality of vertebral segment development due to various causes, and congenital scoliosis gradually induces an imbalance in the coronal and/or sagittal planes of the spine during spinal growth, which in turn produces a three-dimensional deformity of the spine (1–3). The incidence of congenital scoliosis in the general population is approximately 1/1,000 to 1/2,000, and the incidence is greatly affected by environmental and genetic factors (4). There are three types of congenital scoliosis (5): Type I: vertebral body formation disorders, including hemivertebrae, butterfly vertebrae, cuneiform vertebrae, etc.; Type II: vertebral body segmentation disorders, including bony bridges, massive vertebra, block vertebrae, etc.; and Type III: mixed type, in which one vertebral body segmentation disorder is combined with a contralateral vertebral body formation disorder. The most common hemivertebral deformity is caused by abnormal unilateral vertebral body formation (6), and fully segmented hemivertebrae and type III congenital scoliosis have early onset and rapid progression (1). Conservative treatment cannot prevent deformity progression, and some researchers recommend early surgical intervention (7–9). Early surgery in young children with congenital scoliosis can slow down or prevent further development of the deformity, thus allowing the unaffected part of the spine to grow normally (2). Although posterior hemivertebra resection combined with pedicle screw internal fixation is effective, children can experience complications such as pedicle fracture and internal fixation prolapse (10–13), and it is difficult to choose suitable internal fixation screws for very young children. Posterior hemivertebra resection without internal fixation can avoid the complications associated with internal fixation, but there are few reports about this kind of treatment. Therefore, this study was performed to evaluate the feasibility and efficacy of posterior hemivertebra resection without internal fixation for congenital scoliosis in very young children.

## Methods

### Patients

This was a retrospective analysis of 16 young children (8 males and 8 females) with congenital scoliosis who were not treated with internal fixation devices after posterior hemilaminectomy at our institution from April 2000 to July 2019, all of whom had type I/III congenital scoliosis. The age at presentation of the patients was a median (interquartile range) of 9.00 (7.75) months (range, 0.5–48 months), and there were 11 cases with clear indications of scoliosis at presentation, 7 cases with an abnormal mass on the back, 4 cases of asymmetry of the lower limbs, 5 cases of abnormal hair on the waist and back, 2 cases of hallux valgus or eversion, and 3 cases of dysuria.

The following tests were completed before surgery: full spine frontal and lateral x-rays in both straight and bent positions to comprehensively assess the static parameters and flexibility of the spine, computer tomography scan and 3D reconstruction to assess the morphology and position of the hemivertebral body and adjacent vertebral bodies as well as the anatomy of the pedicle and posterior vertebral body, magnetic resonance imaging and bilateral lower extremity electromyography to exclude neurological disorders, and echocardiography to assess relevant congenital anomalies.

### Study methods

All operations were performed by the same specialist with extensive experience in the treatment of congenital scoliosis. The operative site, operative time, intraoperative blood loss, and follow-up time were collected; and the Cobb angles of the segmental curve, main curve, proximal compensatory curve,distal compensatory curve, and segmental kyphosis were measured on preoperative, postoperative, and final follow-up frontal and lateral radiographs of the children.

### Surgical procedure

All patients underwent posterior hemivertebrectomy with unilateral hemivertebra exposure in type I scoliosis and bilateral exposure in type III. No spinal instrumentation was used after simple hemivertebra resection.

Under general anesthesia, the patient was placed in the prone position. Positioned with the C-arm and marked, the posterior aspect of the spine was exposed unilaterally at the level of the hemivertebrae and adjacent vertebrae using a standard median incision. The paravertebral muscles were stripped to expose the small joints, laminae, and spinous processes above and below the hemivertebral body, and the extranodal and anterior periosteum of the hemivertebral body were stripped. The posterior portion of the ipsilateral hemivertebral body was excised, including the laminae with transverse process, the facet joints, and the posterior part of the pedicle of the vertebral arch. If the hemivertebra was located in the thoracic segment, the posterior corner of the rib connected with the hemivertebra to the part of the rib capitulum was also removed taking care to avoid damaging the parietal pleura, and the broken end of the rib was closed with bone wax. In the event of epidural bleeding during the operation, bipolar electrocoagulation hemostasis was performed. The spinal cord and the nerve roots above and below the hemivertebrae were carefully separated and protected with cotton sheets, and the residue of the hemivertebral body and adjacent intervertebral discs was scraped off. The endplates of the convex side of the vertebral body were removed up to the cancellous bone, and the opposite end plate was preserved. Autologous and allogeneic bone blocks were implanted at the hemivertebral body resection. In cases of contralateral bone bridging, unilateral exposure was inadequate and bilateral exposure was required to sever the bone bridge and remove the fused rib head. Motor evoked potential and somatosensory evoked potential were monitored during the operation.

An orthopedic brace or plaster vest was applied immediately after surgery. For infants who could not walk, they stayed in bed with braces or plaster vests for 3 weeks after surgery, and after that they could sit and be held upright. For children who could walk, they could get out of bed and walk three weeks after surgery, but strenuous exercise was to be avoided. All children wore an orthopedic brace or plaster vest all day for the first 3 months after surgery. After 3 months, if osseous fusion had occurred, the brace could be worn during the day and removed at night. During the 6-month follow-up period, the plaster and orthopedic braces were timely adjusted or replaced according to the growth rate of the children.

### Statistical analysis

SPSS 25.0 software was used for statistical analysis. Preoperative, postoperative, and final follow-up data were analyzed by the Wilcoxon signed-rank test or paired *t*-test. Data that had a normal distribution are presented as the mean ± standard deviation, and measures that did not conform to a normal distribution are presented as the median (interquartile range.). Differences were considered statistically significant at *P* < 0.05.

## Results

The 16 children (8 males and 8 females) all had type I/III congenital scoliosis with a single hemivertebral deformity, and 7 cases were combined with spinal cord tethering syndrome, 2 cases with spinal cord cavity, 5 cases with spinal cord longitudinal bifida, 3 cases with spondylolisthesis, and 1 case with spina bifida. A total of 16 hemivertebrae were resected, of which 3 were located in the main thoracic segment (T6–T9), 9 in the thoracolumbar segment (T10–L2), 3 in the lumbosacral segment (L3–S1), and 1 in the sacral segment (S2–S5). The age at surgery was 9.00 (7.75) months (range, 0.5–48 months), the mean postoperative follow-up time was 94.31 ± 65.63 months (range, 36–222 months), the mean operative time was 119.88 ± 37.93 min (range, 65–195 min), and the mean bleeding volume was 95.6 ± 56.78 ml (range, 40–250 ml) (Table 1).

**Table 1:** Clinical data of the included patients.

Case	Age (months)	Sex	Hemivertebra position	Type	Operation time (min)	Volume of bleeding (ml)	Follow-up time (months)	Final correction rate of the segmental curve (%)
1	10	M	T8	I	120	80	197	47.2
2	6	F	T10	I	160	150	48	75.0
3	3	M	S1	I	85	60	55	93.5
4	4	F	T9	III	120	40	36	69.0
5	18	M	L2	I	75	60	39	70.8
6	5	F	L1	III	120	100	38	66.0
7	0.5	M	L5	I	65	50	145	77.4
8	12	M	L3	I	65	50	191	84.4
9	5	F	L2	I	165	120	126	−13.5
10	48	F	T11	III	195	100	55	61.7
11	30	F	S2	I	135	120	222	90.9
12	12	M	T11	I	115	70	127	44.7
13	2	M	L1	I	142	60	38	52.3
14	9	F	T10	I	113	175	36	93.3
15	11	F	T11	I	88	50	108	−5.6
16	9	M	T6	I	155	250	48	86.2

M, male; F, Female.

### Coronal plane

**Figure 1:**
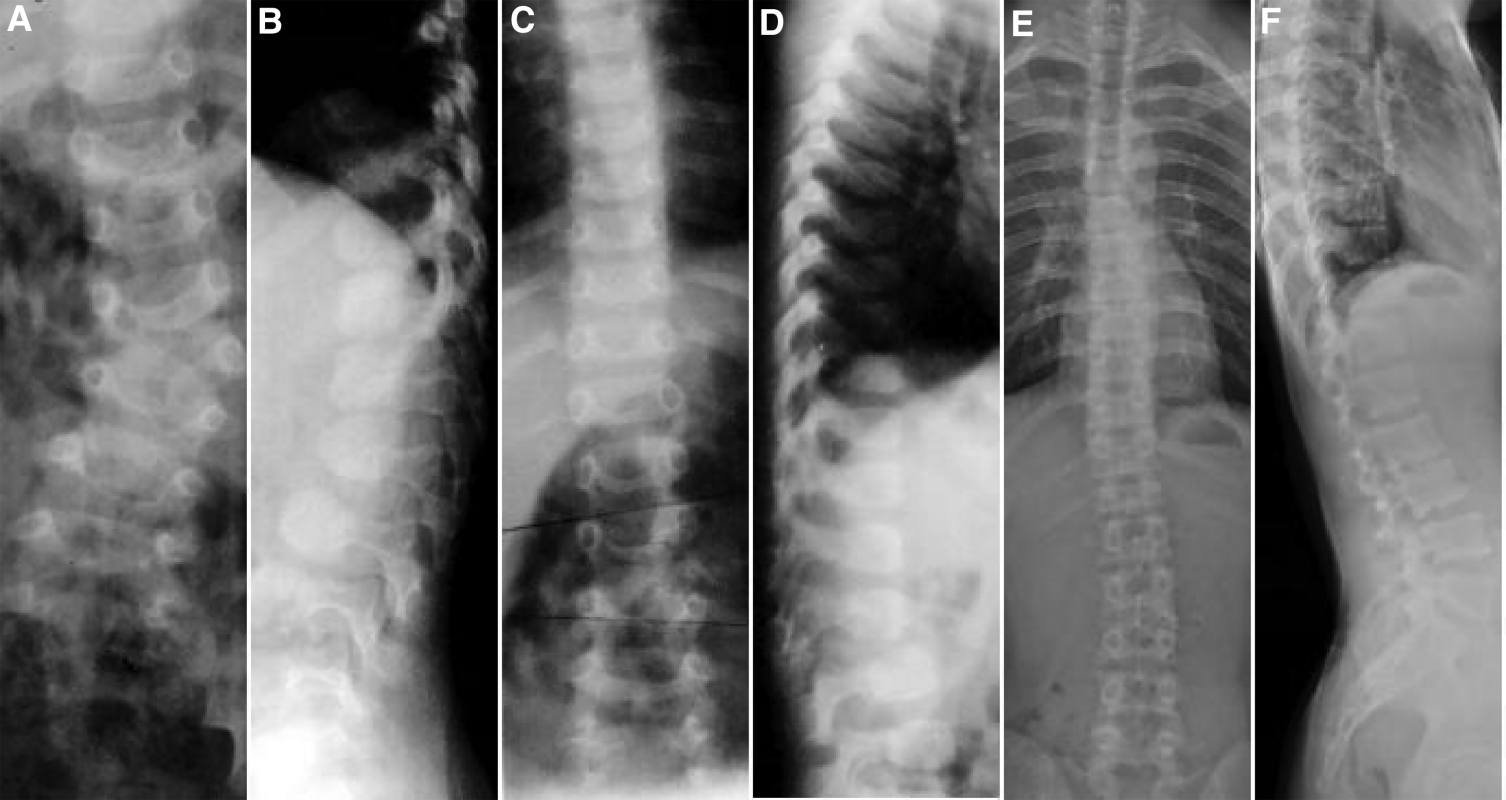
Case 8, Male, 12 months old. Preoperative orthogonal (**A**) and lateral (**B**) x-ray images showing the left hemivertebrae of L3. Intraoperative hemivertebral resection was performed *via* the posterior approach, and postoperative orthogonal (**C**) and lateral (**D**) x-rays are shown. At the follow-up at 15 years and 11 months, the orthogonal (**E**) and lateral (**F**) x-rays showed satisfactory correction of the coronal and sagittal deformity with no loss of curve correction.

The differences between the preoperative Cobb angle of the segmental curve (39.50 ± 9.70°) and the postoperative (19.19 ± 8.56°) and final follow-up (14.94 ± 12.11°) angles were both statistically significant (*P *< 0.001 and *P* = 0.001, respectively) (Table 2). The mean correction rates postoperatively and at final follow-up were 49.98% and 62.09%, respectively. During the follow-up period, 12 (75%) patients showed sustained improvement in the segmental curve (Figure 1).

**Table 2:** Preoperative, postoperative, and last follow-up radiographic imaging parameters of children with congenital scoliosis who underwent posterior hemivertebral resection without internal fixation.

Parameters	Preoperative	Postoperative	Final follow-up	P (Preoperative vs. Postoperative)	P (Preoperative vs. Final follow-up)
**Coronal plane**					
Segmental curve (°)	39.50 ± 9.70	19.19 ± 8.56	14.94 ± 12.11	<0.001	0.001
Main curve (°)	34.19 ± 14.34	17.00 ± 11.70	17.56 ± 16.31	<0.001	0.005
Proximal compensatory curve (°)	14.88 ± 9.62	7.88 ± 4.66	8.38 ± 8.36	0.002	0.012
Distal compensatory curve (°)	13.50 (10.50)	4.55 (9.25)	5.50 (9.50)	0.001	0.007
**Sagittal plane**					
Segmental kyphosis (°)	10.00 (14.00)	14.00 (24.50)	17.00 (22.55)	0.109	0.408

The data are shown as the mean ± standard deviation or as the median (interquartile range).

**Figure 2:**
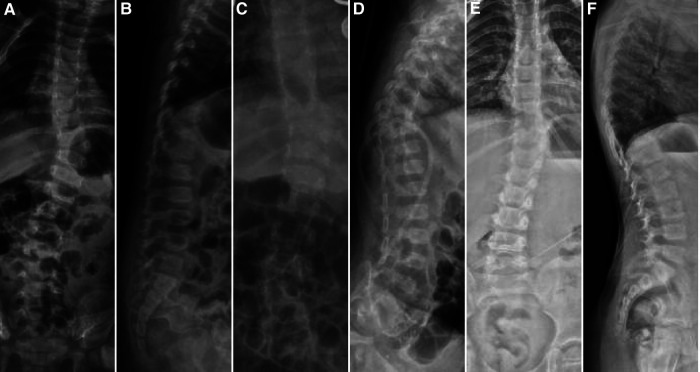
Case 14, female, 9 months old. Preoperative orthogonal (**A**) and lateral (**B**) x-rays showing the left hemivertebrae at T10. Intraoperative hemivertebra resection was performed *via* the posterior approach, and postoperative orthogonal (**C**) and lateral (**D**) x-rays are shown. At 3 years of follow-up, the orthogonal (**E**) and lateral (**F**) x-rays showed good correction of the coronal and sagittal deformity.

The differences between the preoperative Cobb angle of the main curve (34.19 ± 14.34°) and the postoperative (17.00 ± 11.70°) and the last follow-up (17.56 ± 16.31°) angles were both statistically significant (*P* < 0.001 and *P* = 0.005, respectively) (Table 2). The mean correction rates postoperatively and at last follow-up were 53.22% and 46.93%, respectively, and in 11 (68.75%) cases the patients showed sustained improvement of the principal curve during the follow-up period (Figure 2).

The differences between the preoperative Cobb angle of the proximal compensated curve (14.88 ± 9.62°) and the postoperative (7.88 ± 4.66°) and the last follow-up (8.38 ± 8.36°) angles were statistically significant (*P* = 0.002 and *P* = 0.012, respectively), and the mean correction rates postoperatively and at last follow-up were 34.46% and 33.89%, respectively. The differences between the preoperative Cobb angle of the distal compensated curve 13.50° (10.50°) (range, 4°–30°) and the postoperative 4.50° (9.25°) (range, −3° to 25°) and last follow-up 5.50° (9.50°) (range, −3° to 33°) angles were statistically significant (*P* = 0.001 and *P* = 0.007, respectively), and the mean postoperative and last follow-up correction rates were 61.11% and 51.88%, respectively (Table 2).

### Sagittal plane

There was no statistically significant difference in the preoperative Cobb angle 10.00° (14.00°) (range, −31° to 41°) for segmental kyphosis compared with the postoperative 14.00° (24.50°) (range, −6° to 46°) and the final follow-up 17.00° (22.55°) (range, −40° to 56°) angles (*P* = 0.109 and *P* = 0.408, respectively), and the mean postoperative and last follow-up correction rates were 2.44% and 51.86%, respectively (Table 2).

### Complications and reoperation

**Figure 3:**
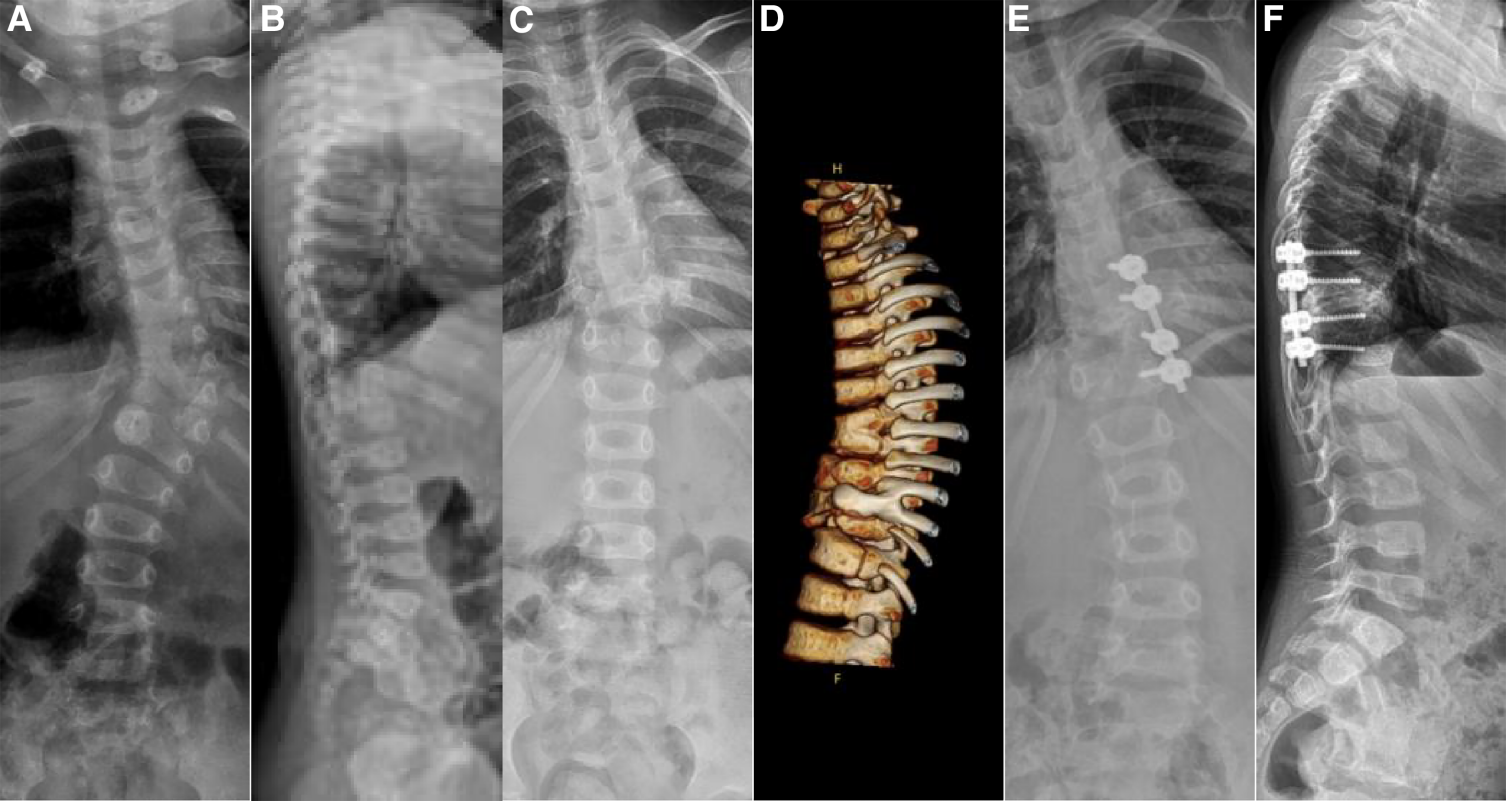
Case 2, female, 6 months old. Preoperative orthogonal (**A**) and lateral (**B**) x-ray showing the left hemivertebrae of T10. Intraoperative hemivertebra resection was performed *via* the posterior approach. After 4 years of follow-up, orthogonal x-ray (**C**) and lateral computer tomography (**D**) showed good correction of coronal scoliosis, but sagittal kyphosis was aggravated, and a second internal fixation revision surgery was performed. Post-revision orthogonal (**E**) and lateral (**F**) x-ray showed good correction of the coronal and sagittal deformity.

**Figure 4:**
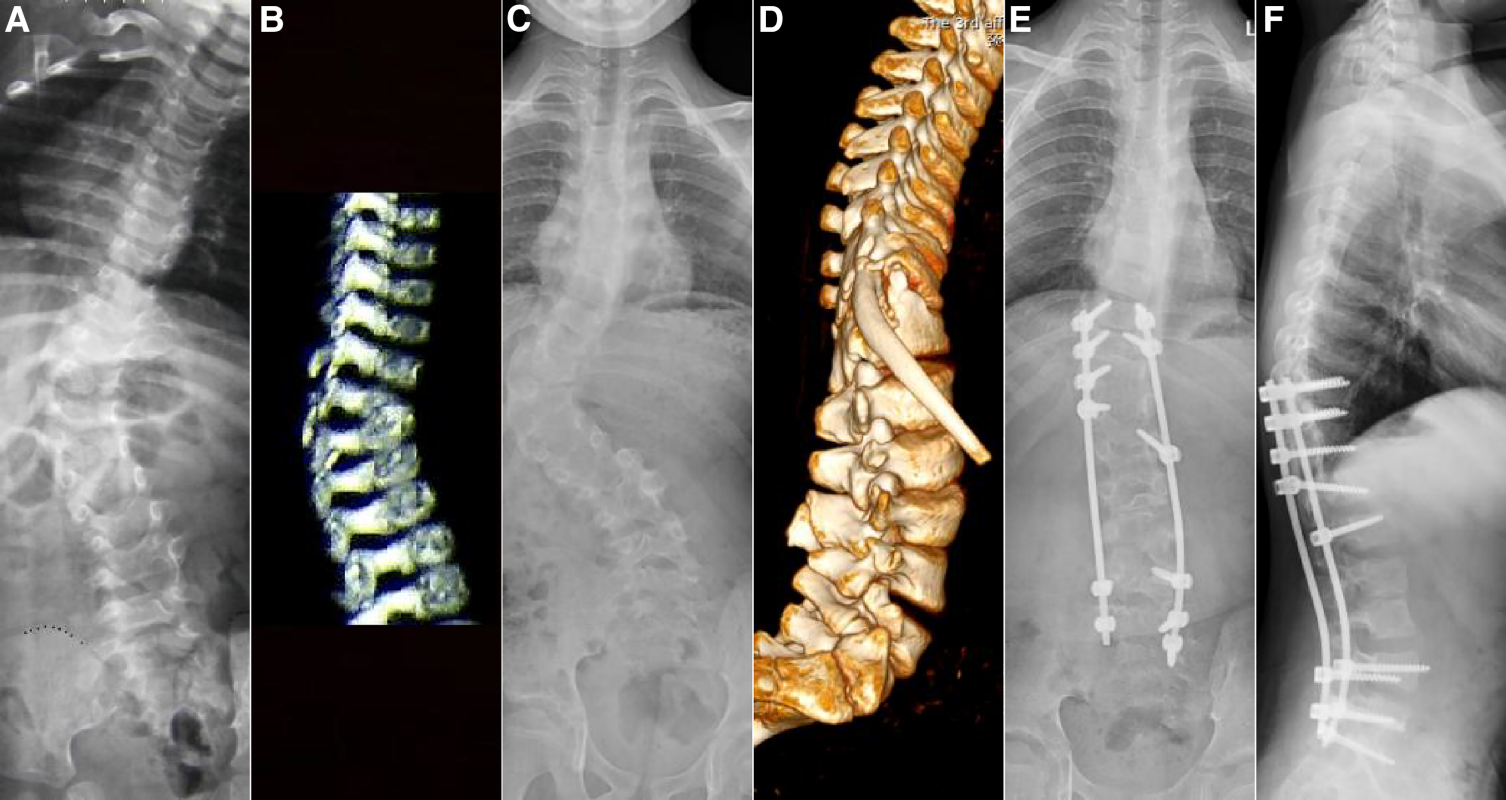
Case 15, female, 11 months old. Preoperative orthogonal x-ray (**A**) and lateral computer tomography (**B**) showing the right hemivertebrae of T11. Intraoperative hemivertebra resection was performed *via* the posterior approach. After 9 years of follow-up, orthogonal x-ray (**C**) and orthogonal computer tomography (**D**) showed local pseudoarticular formation and worsening deformity, and a second internal fixation revision surgery was performed. Post-revision orthogonal (**E**) and lateral (**F**) x-ray showed good correction of the deformity in the coronal and sagittal planes.

There were no exacerbations of neurological symptoms, no infections, and no cerebrospinal fluid leakage after surgery in any patient. During the follow-up period, 3 cases had loss of curve correction due to residual hemivertebrae: 1 case (case 2) had increased sagittal segmental kyphosis (Figure 3), 1 case (case 9) had progressive aggravation of the proximal compensatory curve, and 1 case (case 12) had progressive aggravation of the main curve. In addition, 1 case (case 15) had local pain due to local pseudarthrosis formation (Figure 4), and scoliosis was progressively aggravated at the follow-up. All of the patients with complications were treated with secondary internal fixation surgery.

## Discussion

Hemivertebral deformity is one of the most common causes of congenital scoliosis, and its severity depends on four main factors, namely the type, the location and number of hemivertebrae, their relationship with each other, and the age of the patient (2, 14). Most hemivertebrae have normal growth plates, especially fully segmented and non-integrated hemivertebrae, and thus have growth potential similar to that of normal vertebrae (2, 15), and therefore the scoliosis deformity progressively worsens with further spinal growth. If left untreated, the principal curve will gradually increase to more than 41° in approximately 85% of patients (15). For scoliosis caused by hemivertebrae, conservative treatment such as bracing is not effective, so surgery is often used for orthopedic treatment (16, 17).

Surgical modalities for the treatment of congenital scoliosis include posterior *in situ* fusion, anterior-posterior convex epiphyseal block, subcutaneous bracing on the concave side of the convex epiphyseal block, and hemivertebrectomy (18). For congenital scoliosis secondary to hemivertebral deformity, posterior hemivertebrectomy is preferred (10, 19, 20). The age at which the patient should undergo surgery is controversial, but there is a greater consensus that surgery before 3 years of age yields better outcomes (1, 14, 19, 21, 22). The goal of early surgery is to maximize the correction of the deformity, prevent the progression of scoliosis, shorten the fused vertebral segments, and reduce the impact on spinal growth before decompensatory structural changes can occur in the spine (23). Young children with good flexibility and only mild primary deformities require only short-segment fusion, which is less difficult to perform, poses less risk of neurological injury, and can achieve better immediate orthopedic results (22). In this study, the median age of the 16 children at surgery was 9 months, and the results at the last follow-up showed that the mean angular correction rates of the segmental and principal curve in the coronal plane were 62.09% and 46.93%, respectively. A total of 12 (75%) cases of segmental curve and 11 (68.75%) cases of main curve saw continuous improvement, which indicated that the children in this age group could tolerate the operation and obtain a certain corrective effect in the coronal plane of the spine, thus avoiding further aggravation of the deformity in most of the children studied here.

Hemivertebrectomy with short segment fixation and fusion has now become the mainstream treatment for congenital scoliosis caused by a single hemivertebra, and the application of pedicle screws can significantly improve the correction rate of congenital scoliosis (24, 25). Although studies have demonstrated that the pedicle screw system is safe and effective in pediatric patients (12, 26), the pedicle is not well developed in very young children (11, 25). Especially for children under 5 years old, it is difficult to maintain a good internal fixation position (20), and the failure of internal fixation implantation is a challenge for hemivertebrectomy in young children (27), mainly including complications such as pedicle fracture and internal fixation prolapse (10–13). In 2003, Ruf et al. (12) reported that among children under 6 years of age who underwent posterior hemivertebrectomy with transpedicular instrumentation, 3 out of 28 had failed internal fixation and 2 out of 28 had pedicle fractures. In 2013, Wang et al. (27) reported that of 36 children with a mean age of 4 years and 11 months, 2 had pedicle overload and fracture, suggesting revision surgery. A study by Guo et al. (11) in 2016 showed a complication rate of 9.5% in 116 posterior hemivertebrectomies, of which 63.6% were related to implantation. Other reports in the literature have shown that congenital scoliosis revision surgery is mostly due to the failure of internal fixation (27) or the inappropriate choice of surgical approach (28). Very young children are sometimes less likely to have access to well-suited internal fixation devices because of their own bone developmental characteristics (20). Because there are no suitable internal fixation screws for use in these very young patients, internal fixation cannot be applied to these children who require treatment. Therefore, the most effective fixation after hemivertebrectomy in order to avoid further worsening of the deformity in younger children with congenital scoliosis needs to be determined.

In 1945, Smith–Peterse (29) first reported the use of posterior lumbar osteotomy for spinal deformities with good postoperative results using plaster undershirt fixation, and this provided a theoretical basis for not using internal fixation after posterior hemivertebrectomy in younger children. Our results showed that early posterior hemivertebrectomy without internal fixation in younger children achieved certain therapeutic results while avoiding complications related to internal fixation, and continued postoperative orthopedic bracing helped to maintain the orthopedic effect and control the progression of the deformity and no complications related to spinal cord injury were observed during the longer follow-up. The results of this study also showed that this surgical approach was effective in correcting coronal deformities, and the difference was statistically significant, but the correction of sagittal kyphosis was not significant. In fact, the thoracic and thoracolumbar kyphosis tended to be aggravated during the follow-up period, especially the thoracolumbar kyphosis, suggesting that the operation may not be suitable for thoracic and thoracolumbar hemivertebral deformities. Four of the patients underwent secondary surgical treatment during follow-up, and three of them (cases 2, 9, and 12) lost their orthopedic effect due to incomplete removal of the hemivertebrae and one (case 15) had an increased deformity in the coronal and sagittal planes due to the formation of local pseudarthrosis. Therefore, complete resection of the bone from the half vertebral body to the upper and lower vertebral bodies was necessary to prevent the aggravation of the deformities and the formation of pseudarthrosis.

## Limitations

The small number of patients in this study limited the comparison and statistical analysis of surgical outcomes between different vertebral segments. Furthermore, some of the very young patients did not cooperate well with postoperative treatment, which may have influenced the curve correction during the follow-up period. In addition, the follow-up time in some cases was short and the curve correction of these children may change in future follow-up. Increased numbers of cases and the extension of follow-up time will be helpful in further evaluating the long-term effect after surgery.

## Conclusion

In conclusion, posterior hemivertebral resection without internal fixation is a feasible treatment for type I/III congenital scoliosis in very young children. The exposure of posterior, unilateral, and short segments during the operation has the advantages of less trauma and less bleeding, which can effectively delay or prevent the development of more serious structural deformities of the spine, maintain the range of motion of the spine, and effectively reduce hospitalization costs and internal fixation-related complications. Postoperative bracing or several plaster fixations are required to assist in the orthopedic process, resulting in higher correction rates and delaying the age of spinal fusion surgery. This procedure is particularly suitable for lumbosacral and sacral hemivertebral deformities, but it is less effective for thoracic deformities, especially thoracolumbar hemivertebral deformities, and further improvement of postoperative external fixation is needed.

## Data Availability

The raw data supporting the conclusions of this article will be made available by the authors, without undue reservation.
